# *In Vitro* Study of Potentially Probiotic lactic Acid Bacteria Strains Isolated From Traditional Dairy Products

**DOI:** 10.5812/jjm.10168

**Published:** 2014-06-01

**Authors:** Hooshang Niazi Amraii, Hamid Abtahi, Parvaneh Jafari, Hamid Reza Mohajerani, Mohammad Reza Fakhroleslam, Neda Akbari

**Affiliations:** 1 Microbiology Department, Science Faculty, Science and Research Branch, Islamic Azad University, Arak, IR Iran; 2 Molecular and Medicine Research Center, Arak University of Medical Sciences, Arak, IR Iran

**Keywords:** Probiotic, Lactobacillus, Bifidobacterium

## Abstract

**Background:**

Probiotic microorganisms are selected based on their long history of use as well as their lack of side effects. Nowadays, the consumption of probiotic products is growing intensively in developing countries. Researchers who work in the food industry and research centers pay more attention to the identification of new probiotic bacteria with better performance characteristics as well as investigation of their performance because these findings can be very effective in promoting sale and consumption of these products.

**Objectives:**

Hence, this study was performed following these objectives: isolating indigenous lactic acid bacteria from traditional dairy products in Markazi Province, screening strains with probiotic characteristics, identifying strains and performing microbial collection of probiotic strains with indigenous potential.

**Materials and Methods:**

In this study, the samples were screened from traditional dairy products, such as fresh yogurt, curd, Tarhana and Ghareghoroot of Markazi Province villages. Samples were enriched in de Man, Rogosa and Sharpe (MRS) broth, and different strains were isolated and purified from this culture on MRS agar medium. Isolated strains were investigated by microscopic observations, considering the following factors: catalase capability, resistance to acid and bile, bile salt hydrolysis and antibiotic susceptibility pattern.

**Results:**

Nineteen Gram-positive and catalase-negative strains belonging to the *Lactobacillus* genus were isolated from the above-mentioned diary samples. Seven strains were resistant to acid and bile in which acid resistance was between 21.08% and 122.33% and bile resistance was between 94.08% and 152.93%, respectively. All isolated strains were susceptible to different antibiotics and a small percentage had the ability to hydrolyze Sodium Taurocholate.

**Conclusions:**

There are many of different species of *Lactobacillus* probiotics in traditional dairy products of the Markazi province, based on the findings of this study. It is recommended for researchers to isolate these strains and investigate their probiotic characteristics in order to reproduce them for use in food production as well as for medical treatment.

## 1. Background

Great attention is currently drawn to probiotics, prebiotics or their combined use as synbiotics, to improve human health via natural sources. Probiotics are defined by the FAO/WHO as “live microorganisms that, when administered in adequate amounts, confer health benefits on the host” (1). Probiotics have become a major focus of lactic acid bacteria (LAB) research over the past 10 years, with most attention drawn to the genera *Lactobacillus* and *Bifidobacterium* (2). These organisms have been widely reported to exert many beneficial effects, such as activation of the immune system, prevention of cancer cell growth, maintenance of mucosal integrity and presentation of an antagonistic environment for pathogens (2, 3). There has been an increase of interest regarding the commercial utilization of *Lactobacillus* strains isolated from traditional and naturally fermented dairy products, which possess health-promoting effects. Research on *Lactobacilli* isolated from such traditional and naturally fermented dairy products reveals a long history of safe use (2, 4, 5).

Nowadays, a variety of microorganisms, typically food grade lactic acid bacteria (LAB) and *bifidobacteria* have been evaluated for their probiotic potential and are applied as adjunct cultures in various types of food products or therapeutic preparations (5). To be beneficial to human health, a probiotic must fulfill several criteria; for instance, it must survive passage through the upper gastrointestinal tract (GIT) and must be able to function in the gut environment (1, 4, 6). Probiotics also need to possess the ability to survive and be viable in the products, during food production and storage (1, 7). Therefore, a proper evaluation of each candidate probiotic strain becomes an important step for further implementation as a culture adjunct. The most commonly used organisms in probiotic preparations are the LAB. Therefore, the following sections will be devoted to general information, taxonomy and importance of food with this important group of bacteria (1, 8). However, to provide health benefits, *Lactobacillus* strains, which are mostly delivered in a food system, must overcome physical and chemical barriers in the gastrointestinal tract, especially acid and bile stresses, and have antagonistic activity against bacterial pathogens (2).

## 2. Objectives

Due to the fact that the Markazi Province is known for its abundant production of traditional dairy products, microbial collections of probiotic strains of lactic acid bacteria was performed in this area (ensuring safety of isolated strains (GRAS)).The aim of the current study was then to investigate the probiotic characteristics of these collected strains. This attempt was a step towards addressing the increasing importance of these products as well as resolving our dependence on these microbial sources.

## 3. Materials and Methods

### 3.1. Isolation of Strains of Lactic Acid Bacteria From Traditional Dairy Products

In this study, all chemicals and cultivation mediums were provided by Merck Co. Firstly, for isolation of the strains, samples of traditional dairy products, including yogurt, curd, Tarhana and Ghareghoroot, were collected from the Markazi province. Secondly, the collected samples were transported to the laboratory after coding. Thirdly, samples were enriched using falcons containing de Man, Rogosa and Sharpe Broth medium in an incubator at 37 ºC with atmospheric carbon dioxide. Tarhana and curd samples have hard tissues, hence cannot be easily mixed with the cultivation medium. Thereby, these samples were firstly dissolved in sodium citrate and were then added to MRS broth medium (9). After enrichment, bacteria were cultivated in MRS agar and incubated as before (i.e. cultivation in MRS Broth medium). Grown strains were isolated based on colony morphology and catalase capability and were then coded (Table 1). Finally, isolated strains were kept in 50% glycerol at 40 ºC until complementary tests were performed.

**Table 1.** Antibiotics Susceptibility of *Lactobacillus* sp. Isolated From Dairy Products; Yogurt (Y), Tarhana (T), Ghareghoroot (GH)

Sample	Nalidixic Acid	Clindamycin	Chloramphenicol	Ampicillin	Tetracycline	Erythromycin
**Y2**	S	S	S	S	S	S
**Y3**	S	S	S	S	S	S
**Y4**	S	S	S	S	S	S
**Y5**	S	S	S	S	S	S
**Y6**	S	S	S	S	S	S
**T**	S	S	S	S	S	S
**GH**	S	S	S	S	S	S

### 3.2. Investigating Resistance to Acid

First, the pH of the MRS Broth medium was adjusted to pH of two with hydrochloric acid. Then, the medium was inoculated with a certain amount of bacteria suspension for two hours at 37 °C. In the next step, the cells were isolated using centrifugation at 6000 rpm for 10 minutes and inoculated in MRS Broth cultivation medium. The optical absorption of the samples was determined to be 600 nm. Resistance percentage of strains to stomach acid was determined by comparing optical absorption of the samples with the control sample (pH = neutral). It should be noted that this test was repeated three times for each sample (10).

### 3.3. Investigating Resistance to Bile

The isolated strains were cultivated in medium containing 0.2% bile salts. The level of strains resistance was determined by comparing optical absorption of the sample with the control sample (cultivation medium without bile salts) (Diagram 2). It should be noted that this test was repeated three times for each sample (10).

### 3.4. Bile Salts Hydrolysis Test

For this test, the isolated strains were cultivated in medium containing 0.2% sodium taurocholate. The medium was incubated to allow dissolving. The incubation was performed for 48 to 72 hours at 37 ºC in an incubator containing CO_2_. Then, plates were examined for white precipitates. This white precipitate was a sign of bile salt hydrolysis (10).

### 3.5. Investigating Antibiotic Resistance

In order to determine antibiotic resistance, first 1% of strains isolated from newly cultivated samples were added to warm sterile MRS agar with half concentration of MacFarlane. Then, a fixed amount of the medium was divided into plates. After the cultivation medium was made (i.e. became solid) antibiotic disks were placed at regular intervals on plates and incubated in a CO_2_ incubator at 37 ºC. After 24 hours, each plate was retrieved and measured using a metric rule for the area of inhabitation (11).

### 3.6. Identification and Taxonomical Studies

The isolated strain was identified according to methods described in Bergey's Manual of Determinative Bacteriology (12). Also, 16S rDNA sequence analysis was performed for phylogenetic recognition of the new strain.

## 4. Results

**Figure 1.**
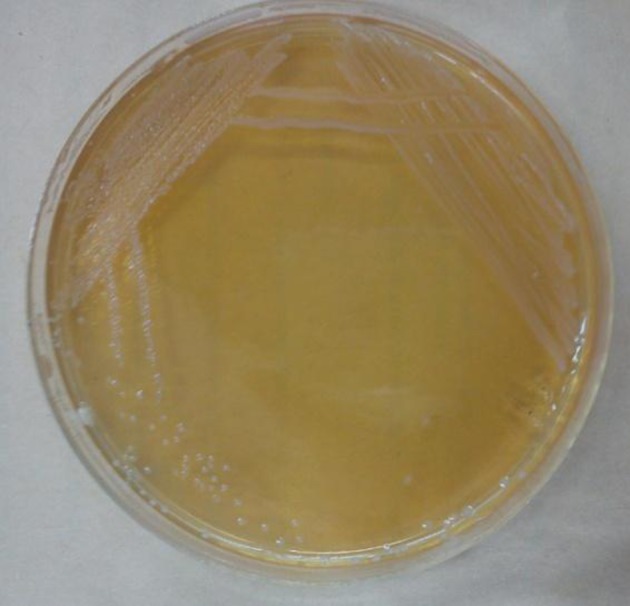
The Colony Isolated From Yogurt

**Figure 2.**
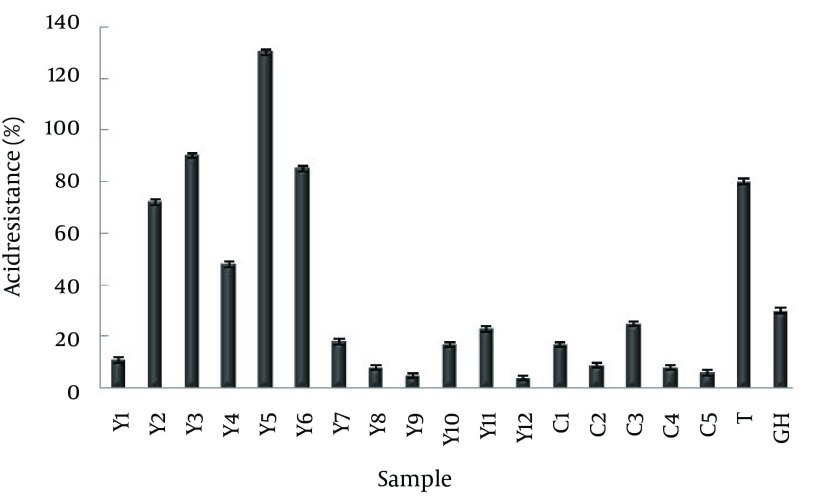
Acid Tolerance of *Lactobacillus* sp. Isolated From Dairy Products; Yogurt (Y), Tarhana (T), Ghareghoroot (GH), Curd (C)

Among numerous isolated bacteria, 19 strains were isolated from dairy products (Figure 1), which were all Gram-positive and catalase-negative. Based on biochemical and physiological tests performed according to Bergey's Manual of Systematic Bacteriology, the strain was identified as *Lactobacillus* sp. (12). Using BLAST analysis of 16S rDNA sequences, the nucleotide sequence was found to be identical to parts of the *Lactobacillus crustorum*, *L. plantarum* and *L. paracasei* genomes. Probiotic bacteria should pass through the highly acidic stomach in order to reach the intestine and create proper conditions for residence. Therefore, the first step in screening of probiotic *Lactobacilli* strains is selecting those that are acid resistant (Figure 2).

**Figure 3.**
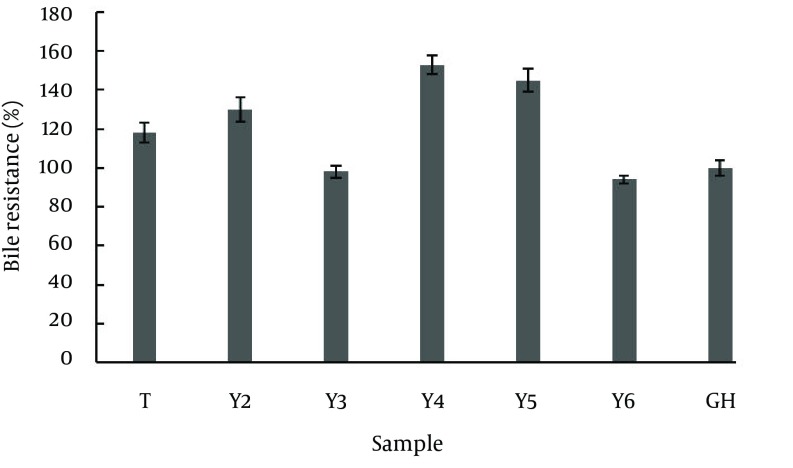
Bile Tolerance of *Lactobacillus* sp. Isolated From Dairy Products; Yogurt (Y), Tarhana (T), Ghareghoroot (GH)

**Figure 4.**
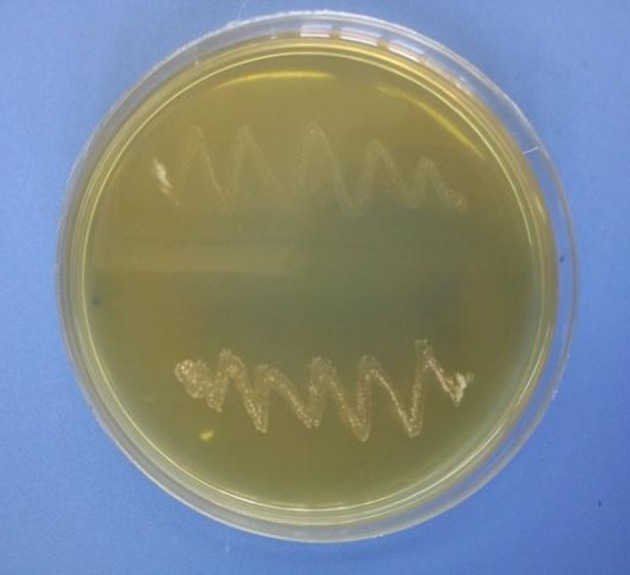
Bile Salt Hydrolysis in Sodium Taurocholate Medium by T And Y2 strains

Probiotic bacteria should not only be tolerant to acidic conditions of the stomach but also be resistant to intestinal bile salts, so that they could survive in the digestive system. In this study, Y2, Y3،Y4, Y5, Y6, T and GH, which were resistant to highly acidic conditions, were evaluated for their ability to grow in the presence of 0.2% bile salts; all strains were highly resistant to bile salts (Figure 3). The results showed that all of the selected strains were susceptible to antibiotics used in this test (Table 1). The results showed that among isolated strains Y2, Y3, Y4, Y5, Y6, T and GH were positive regarding bile salt hydrolysis (Figure 4).

## 5. Discussion

It has been known for years that dairy products are important for human health. One of the main problems of probiotic bacteria in dairy products is their intolerance to products’ ecological conditions (1-3). Moreover, their undesirable taste and odor is another problem that should be considered. In the study conducted by Coeuret et al. on traditional unpasteurized milk and cheese in France during 2004, 88 strains of *Lactobacilli* were isolated from these two products from which only four strains had probiotic potential (13). In another study on 122 *Enterococcus Faecium* strains isolated from traditional cheese of Argentina, 11 strains were able to grow in acidic conditions and bile salts (9).

In order to evaluate their beneficial effects in the host, probiotic bacteria must transit through the highly acidic stomach conditions in order to reach the intestine. Thus, resistance towards low pH is essential for the survival of the probiotics. As shown in Diagram 1, the most acid-tolerant strains were respectively isolated from yogurt, Tarhana and Ghareghoroot; however, no resistant strains were obtained from curd. The results indicated that the best option for isolation of potentially probiotic *Lactobacilli* is from yogurt which was obtained from Nazmabad and Moradabad villages.

Another key characteristic of probiotic bacteria is their resistance and ability to grow in the presence of bile salts in order to survive in the digestive system. Gilliland et al. performed extensive studies on Lactobacilli susceptibility, isolated from dairy products, to bile salts as well as the importance of susceptibility determination in selection of probiotic bacteria (14, 15). They showed, in their following studies, that tolerance to bile salts differs among strains of Lactobacillus species isolated from the intestine. Growth and reproduction of bacteria in the intestines of cattle is dependent on their bile tolerance (16).

Gilliland et al. used a 0.3 concentration of oxgall bile salt in his studies, which was a mixture of cholic acid (bile salts insoluble in water) and taurocholic acid (bile salts dissolved in water). This mixture (oxgall) is most similar to human bile salts. The basis of his studies was determination of the difference between lag phase of bacterial growth in the presence and absence of bile salts (17). Similar studies were done by Liong and Shah (2005), Lin, et al. (2007) and Pennacchia et al. (2004) in which the same methods were used to evaluate bile salt tolerance of Lactobacillus strains (18-20). In this study, bile salt tolerances of selected acid resistant strains were investigated. Seven acid resistant strains, considering their ability to grow in the presence of 0.2% bile salts, were evaluated. All of the stains were highly resistant to bile salts, where maximum resistance was equal to 152.93%. From our experiments, we found that isolated *Lactobacillus* sp. show a broad range of resistance to most antibiotics including chloramphenicol. This may be possibly due to the wide use of antibiotics in veterinary medicine and agriculture.

Biochemical characterization of strains showed that resistance and tolerance to bile salts are not dependent on species; however, they are different amongst strains of the same species. This extensive range of resistance to bile salts and diversity of resistance in different strains observed in this study was reported by other researchers including, Liong and Shah (2005), Pennacchia (2004), Mishra and Prasad (2005) (18, 20, 21). These results indicate that lactic acid strains, which are resistant to acidic conditions and bile salts, isolated from traditional fermented dairy products in Iran, could be good candidates for use along commercial primers in order to enhance health of dairy products.

In conclusion, this study showed that direct screening methods with inhibition of acid-sensitive bacteria growth could be an appropriate method for isolation of potential probiotic strains. Moreover, this method facilitates investigation of probiotic characteristics of dairy products. Amongst the four different samples of traditional dairy products, 19 Lactobacillus strains were isolated, from which seven strains were able to survive at pHof2 and grow in 0.2% bile salt. The results showed that yogurt and Tarhana of Nazmabad and Ghareghoroot of Salehabad had more probiotic potential compared to the other products. Also, biochemical, physiological, morphological and molecular tests showed that Lactobacillus sp. were dominant in traditional dairy products of these regions. Since these isolates were resistant to acid and bile salts, they can be used as potentially probiotic bacteria by promoting host-specific health experiments.

## References

[1] M. Saarela (2000). Probiotic bacteria: safety, functional and technological properties.. J Biotechnol..

[2] Arthure C, Ouwehand AC, Salminen S (2002). Probiotics: an overview of benefical effects. Antonie Van leeuwenhoek..

[3] Chassard C, Grattepanche F, Lacroix C, Kneifel W, Salminen S (2011). Probiotics and health claims: challenges for tailoring their efficacy.. Probiotics and Health Claims..

[4] Ambadoyiannis G, Hatzikamari M, Litopoulou-Tzanetaki E, Tzanetakis N (2005). Probiotic and Technological Properties of Enterococci Isolates from Infants and Cheese.. Food Biotechnol..

[5] Birollo GA, Reinheimer JA, Vinderola CG (2000). Viability of lactic acid microflora in different types of yoghurt.. Food Res Int..

[6] Ouwehand AC, Salminen S, Isolauri E (2002). Probiotics: an overview of beneficial effects.. A Van Leeuw J Microb..

[7] Marteau P, Minekus M, Havenaar R, Huis in't Veld JH (1997). Survival of lactic acid bacteria in a dynamic model of the stomach and small intestine: validation and the effects of bile.. J Dairy Sci..

[8] Fernandez MF, Boris S, Barbes C (2003). Probiotic properties of human lactobacilli strains to be used in the gastrointestinal tract.. J Appl Microbiol..

[9] Saavedra L, Taranto MP, Sesma F, de Valdez GF (2003). Homemade traditional cheeses for the isolation of probiotic Enterococcus faecium strains.. Int J Food Microbiol..

[10] Hoque MZ, Akter F, Hossain KM, Rahman MSM, Billah MM, Islam KMD (2010). Isolation, identification and analysis of probiotic properties of Lactobacillus spp. from selective regional yoghurts.. World J Dairy Food Sci..

[11] Liu C, Zhang ZY, Dong K, Yuan JP, Guo XK (2009). Antibiotic resistance of probiotic strains of lactic acid bacteria isolated from marketed foods and drugs.. Biomed Environ Sci..

[12] Holt JG, Krieg NR, Sneath PHA, Staley JT, Williams ST (1994). Bergey's Manual of Determinative Bacteriology..

[13] Coeuret V, Gueguen M, Vernoux JP (2004). In vitro screening of potential probiotic activities of selected lactobacilli isolated from unpasteurized milk products for incorporation into soft cheese.. J Dairy Res..

[14] Gilliland SE, Speck ML (1977). Deconjugation of bile acids by intestinal lactobacilli.. Appl Environ Microbiol..

[15] Gilliland SE (1979). Beneficial interrelationships between certain microorganisms and humans: Candidate microorganisms for use as dietary adjuncts.. J Food Protect..

[16] Gilliland SE, Staley TE, Bush LJ (1984). Importance of bile tolerance of Lactobacillus acidophilus used as a dietary adjunct.. J Dairy Sci..

[17] Gilliland SE, Walker DK (1990). Factors to consider when selecting a culture of Lactobacillus acidophilus as a dietary adjunct to produce a hypocholesterolemic effect in humans.. J Dairy Sci..

[18] Liong MT, Shah NP (2005). Acid and bile tolerance and cholesterol removal ability of lactobacilli strains.. J Dairy Sci..

[19] Lin WH, Yu B, Jang SH, Tsen HY (2007). Different probiotic properties for Lactobacillus fermentum strains isolated from swine and poultry.. Anaerobe..

[20] Pennacchia C, Ercolini D, Blaiotta G, Pepe O, Mauriello G, Villani F (2004). Selection of Lactobacillus strains from fermented sausages for their potential use as probiotics.. Meat Sci..

[21] Mishra V, Prasad DN (2005). Application of in vitro methods for selection of Lactobacillus casei strains as potential probiotics.. Int J Food Microbiol..

